# Bimodal Presentation Speeds up Auditory Processing and Slows Down Visual Processing

**DOI:** 10.3389/fpsyg.2018.02454

**Published:** 2018-12-05

**Authors:** Christopher W. Robinson, Robert L. Moore, Thomas A. Crook

**Affiliations:** Department of Psychology, The Ohio State University, Newark, Newark, OH, United States

**Keywords:** multisensory perception, auditory processing, visual processing, attention, modality dominance

## Abstract

Many situations require the simultaneous processing of auditory and visual information, however, stimuli presented to one sensory modality can sometimes interfere with processing in a second sensory modality (i.e., modality dominance). The current study further investigated modality dominance by examining how task demands and bimodal presentation affect speeded auditory and visual discriminations. Participants in the current study had to quickly determine if two words, two pictures, or two word-picture pairings were the same or different, and we manipulated task demands across three different conditions. In an immediate recognition task, there was only one second between the two stimuli/stimulus pairs and auditory dominance was found. Compared to the respective unimodal baselines, pairing pictures and words together slowed down visual responses and sped up auditory responses. Increasing the interstimulus interval to four seconds and blocking verbal rehearsal weakened auditory dominance effects, however, conflicting and redundant visual cues sped up auditory discriminations. Thus, simultaneously presenting pictures and words had different effects on auditory and visual processing, with bimodal presentation slowing down visual processing and speeding up auditory processing. These findings are consistent with a proposed mechanism underlying auditory dominance, which posits that auditory stimuli automatically grab attention and attenuate/delay visual processing.

## Introduction

Most of our experiences are multisensory in nature and there is a growing body of research highlighting the complex nature of intersensory interactions (see Stein and Meredith, 1993; Bahrick et al., 2004; Calvert et al., 2004; Spence and Driver, 2004; Spence and McDonald, 2004; Robinson and Sloutsky, 2010a; Talsma et al., 2010; Spence et al., 2012; van Atteveldt et al., 2014; Murray et al., 2016; Spence, 2018, for reviews). For example, when stimuli from different sensory modalities provide complimentary information, bimodal presentation can often facilitate processing and speed up responding (Miller, 1982; Stein and Meredith, 1993; Giard and Peronnet, 1999; Bahrick and Lickliter, 2000; Bahrick et al., 2002; Fort et al., 2002; Colonius and Diederich, 2006; Sinnett et al., 2008).

However, there are many situations where stimuli presented to different sensory modalities are unrelated or even conflict. For example, reading while listening to music or driving while having a conversation both require individuals to simultaneously pay attention to information presented to different sensory modalities. When sensory modalities provide different or conflicting information, stimuli presented to one modality can alter or attenuate processing in the second modality (Colavita, 1974; McGurk and MacDonald, 1976; Shams et al., 2000, 2002; Sloutsky and Napolitano, 2003). If bimodal presentation (e.g., simultaneously presenting auditory and visual information) increases task demands, then processing in both modalities should equally slow down. However, if stimuli in one sensory modality is preferred and/or dominates processing in the other modality, then an asymmetry should be found, with bimodal presentation having no cost on processing in the dominant modality and attenuating processing in the non-dominant modality. The current study contributes to the modality dominance literature by examining how bimodal presentation and task demands affect the speed of discriminating auditory and visual information, and examines if costs are symmetrical or asymmetrical.

Over the last 40 years, most of the research examining modality dominance in adult populations has pointed to visual dominance (Colavita, 1974; Colavita et al., 1976; Posner et al., 1976; Egeth and Sager, 1977; Colavita and Weisberg, 1979; Sinnett et al., 2007, 2008; Koppen et al., 2008; Ngo et al., 2010, 2011; see also Spence, 2009; Spence et al., 2012, for reviews). For example, in the Colavita visual dominance task, participants have to quickly press one button when they hear a tone and a different button when they see a light (Colavita, 1974). Interestingly, participants often press the visual button when both stimuli are presented together (the correct response is pressing both buttons or a third button corresponding to a bimodal stimulus), suggesting that the visual modality dominated encoding and/or responding to multisensory information. Many studies using variations of the Colavita task have weakened but have failed to reverse this effect (see Sinnett et al., 2007; Spence, 2009, 2018; Spence et al., 2012, for reviews, but see Shams et al., 2000, 2002; Robinson and Sloutsky, 2013; Parker and Robinson, 2018, which found auditory dominance using temporal tasks which are better suited for the auditory modality).

A different pattern can be seen in the developmental literature, with simple change detection, McGurk, implicit categorization, Colavita visual dominance, sound induced flash illusion, and induction tasks often showing that infants and children pay more attention to auditory than visual information and/or are more distracted by auditory information (Massaro, 1984; Lewkowicz, 1988a,b; Sloutsky and Napolitano, 2003; Robinson and Sloutsky, 2004, 2010b, 2019; Sloutsky and Robinson, 2008; Nava and Pavani, 2013; Broadbent et al., 2018; see also Robinson and Sloutsky, 2010a; Hirst et al., 2018, for reviews). While auditory and visual dominance effects appear to shift across development (Robinson and Sloutsky, 2004; Nava and Pavani, 2013), many of these studies use vastly different paradigms than the traditional Colavita task (but see Nava and Pavani, 2013), thus, it is unclear different findings across development stem from procedural differences or from developmental differences.

Several recent studies have attempted to address this issue by examining modality dominance in adult populations by using variations of change detection tasks, which are typically used in younger populations (Dunifon et al., 2016; Barnhart et al., 2018). For example, in these studies, adults were sequentially presented with two auditory stimuli, two visual stimuli, or two auditory–visual pairings, and they had to quickly determine if the two stimuli or stimulus pairs were the same or different. Pairing the auditory and visual information together often slowed down visual processing, while having no negative effect on auditory processing. These auditory dominance effects persisted even when adults were told to ignore the auditory information and only respond to the visual information (Dunifon et al., 2016: Experiment 2) and there is some evidence that these effects occur early in the course of processing, with latency of first fixations to the visual stimuli also being delayed when images were paired with auditory information (Dunifon et al., 2016; Barnhart et al., 2018). Thus, while these studies do not use the traditional Colavita task (Colavita, 1974), they do provide evidence suggesting that the auditory stimulus disrupted visual processing, whereas, the visual stimulus had no negative effect on auditory processing.

To account for this asymmetry, it has been suggested that sensory modalities are competing for attentional resources (Robinson and Sloutsky, 2010a; Dunifon et al., 2016; Barnhart et al., 2018; see also Wickens, 1984; Duncan et al., 1997; Eimer and Driver, 2000; Eimer and van Velzen, 2002; Pavani et al., 2004; Sinnett et al., 2007 for related discussions). Moreover, since auditory stimuli are almost always dynamic and transient in nature, these stimuli may automatically engage attention to ensure that they are processed before they disappear. Because attentional resources are finite, resources automatically deployed to the auditory modality should come with a cost - attenuated or delayed visual processing. Thus, auditory stimuli may win the competition because they automatically engaging attention, which may explain why attentional manipulations often fail to reverse auditory dominance (Robinson and Sloutsky, 2004; Dunifon et al., 2016).

However, it is also possible that auditory stimuli may dominate because of high-level mechanisms, or mechanisms that occur later in the course of processing. For example, while visual information quickly decays from iconic memory after a few hundred milliseconds (Sperling, 1960), behavioral and physiological research examining echoic memory often shows that auditory information remains in sensory memory for approximately 2–5 s (Darwin et al., 1972; Lu et al., 1992; but see also Cowan, 1984; Winkler and Cowan, 2005; Nees, 2016). It is possible that the memory trace for the auditory stimulus is stronger than the memory trace for the visual stimulus, due to slower decay of auditory information in sensory memory. Thus, auditory dominance may stem from participants relying on the stronger memory trace when determining if two stimuli are the same or different. Alternatively, it is also possible that the auditory information, especially speech, wins the competition because it is maintained in working memory via rehearsal, whereas, unfamiliar visual images which are difficult to label are more difficult to rehearse (Baddeley and Hitch, 1974).

The primary goal of the present study was to further examine how bimodal stimulus presentation affects speed of auditory and visual processing, and to determine if modality dominance stems from low-level characteristics of the auditory stimulus or from higher-level factors such as slower decay of auditory information and/or from better maintenance of auditory information in working memory. Participants in the current study completed three different bimodal tasks and each task had its own unimodal baselines. In the immediate recognition task, participants were presented with an auditory-visual target stimulus for 1 s, the stimulus pair disappeared for 1 s, and then an auditory-visual test item was presented for 1 s. This is a replication of the Dunifon et al. (2016) and Barnhart et al. (2018) using different classes of stimuli and participants had to quickly determine if the target and test items were exactly the same or different. On immediate recognition trials, competition for attention, differential decay, and better rehearsal of auditory stimuli could all account for auditory dominance (bimodal presentation slowing down visual but not auditory processing).

To determine if differential decay could account for the findings, we ran a delayed recognition task, where we increased the interstimulus duration between the target and test items to 4 s. While researchers disagree on the duration of echoic memory (Darwin et al., 1972; Cowan, 1984; Lu et al., 1992; Winkler and Cowan, 2005; Nees, 2016), increasing the interstimulus interval should increase the likelihood that both auditory and visual information have decayed from sensory memory. Finally, we also introduced a working memory task, which was identical to the delayed recognition task, except that participants were asked to rehearse a six-digit number while determining if the target and test items were the same or different. This manipulation should increase cognitive load and also make it difficult to rehearse the verbal labels. If auditory dominance is still present in the delayed recognition and working memory tasks, this would suggest that the effect is driven by the auditory stimulus quickly engaging attention and delaying/attenuating visual processing. However, if auditory dominance disappears, this would suggest that the effect is probably due to auditory stimuli having a stronger memory trace, with the more robust representation dominating the weaker representation.

## Materials and Methods

### Participants

Forty-eight adults (*M* = 19.38, *SD* = 1.47, 26 female) were recruited from The Ohio State University at Newark through their Introductory Psychology course and received course credit for their participation. All participants in the final sample had normal or corrected to normal vision and hearing (self-reported) and provided consent prior to their participation. An additional four participants were tested but not included in the following analyses due to long periods of time not looking at the computer monitor (*N* = 2), failing to understand the instructions, (*N* = 1), or because of poor discrimination of auditory and visual stimuli when presented unimodally in the immediate recognition task (*N* = 1).

Recruitment and experimental procedures were carried out in accordance with the guidelines and approval of The Ohio State University’s Behavioral and Social Sciences Institutional Review Board, Protocol# 2014B0022, Cross-modal processing across the lifespan. After participants were informed about the nature of the study, they completed an IRB approved informed consent form.

### Apparatus

The task was administered on a Dell Optiplex 3040 desktop computer, and stimulus presentation and rate were controlled using DirectRT (v2016) software. Instructions and visual stimuli were presented on a Dell 1909W monitor with 1440 × 900 resolution and auditory stimuli were presented through Kensington KMW33137 headphones at approximately 65–68 dB.

### Stimuli

**FIGURE 1:**
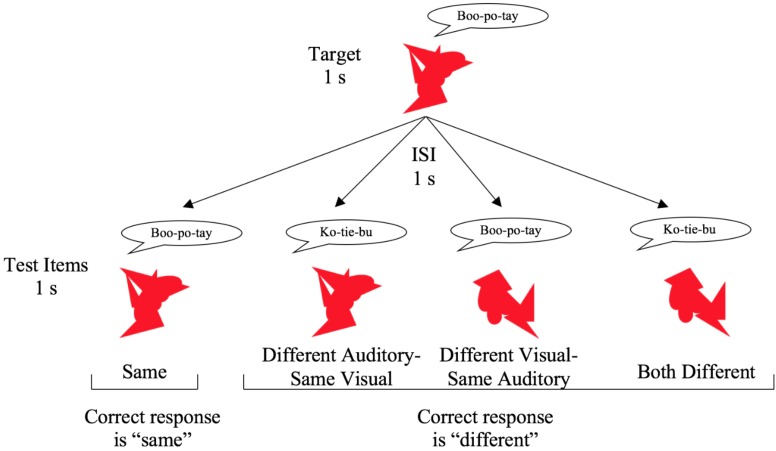
Examples of visual stimuli and overview of the different trial types in the bimodal conditions.

Visual stimuli consisted of 12 novel images, which were created by combining geometric shapes in PowerPoint. All images were monochromatic (red), exported as 300 pixels × 300 pixels bitmaps, and presented centrally on the computer monitor for 1 s. Examples of visual stimuli can be seen in Figure 1. Auditory stimuli consisted of 12 unfamiliar three-syllable words (e.g., “ko-tie-bu,” “boo-po-tay,” etc.). Words were recorded by a male speaker using a Yeti pro microphone. Auditory stimuli were recorded and edited in Audacity and exported as 44.1 kHz wav files. Words were presented at approximately 65–68 dB through headphones and were approximately 1 s in duration (range 0.87–1.03 s). Stimuli in the bimodal conditions were created by simultaneously presenting the unimodal stimuli.

### Procedure

The study contained three different bimodal tasks (Immediate Recognition, Delayed Recognition, and Working Memory) with 26 trials in each task. The first two trials of each block were training trials and they used slightly different stimuli. After the first two training trials, participants were encouraged to ask the experimenter if they had questions about the procedure. Data from the training trials were not included in the analyses, thus, there were only 24 real trials per block. Additionally, each task had its own unimodal baseline consisting of 24 real trials, resulting in six blocks of 24 trials (manipulated within subjects). The order of the six blocks, and trials within each block, were randomized for each participant. The whole procedure took approximately 30 min to complete.

#### Immediate Recognition Task

In the unimodal block, participants were presented with a target for 1 s (either a word or a picture). This was followed by a 1 s Inter-Stimulus Interval (ISI), after which a test item was presented for 1 s (another unimodal stimulus from the same modality as target). Participants were instructed to press 1 on their keyboard if the two words or two pictures were exactly the same or to press 3 if the two words or two pictures were different. Participants were instructed in all tasks/blocks to respond as quickly and as accurately as possible, and that they did not have to wait for the stimulus to disappear before responding. After making a response, participants started the next trial by pressing 1 or 3 on the number pad. This allowed them to control the pace of trial presentation. Unimodal auditory and unimodal visual trials were randomized and intermixed. The task consisted of 12 unimodal auditory trials (6 same and 6 different) and 12 unimodal visual trials (6 same and 6 different).

In the bimodal block, participants were presented with an auditory-visual target using the same stimuli from the unimodal trials. The auditory-visual target was presented for 1 s, followed by a 1 s ISI, and then the auditory-visual test item was presented for 1 s. Participants were instructed to press 1 if the two stimulus pairs were exactly the same and to press 3 if the word, picture, or both word and picture were different. After making a response, participants started the next trial by pressing 1 or 3 on the number pad. As with the unimodal block, there were 24 total trials. Six of the trials were “same” trials where the target and test items were identical. On six of the trials only the word changed (new auditory), on six trials only the picture changed (new visual), and on six trials both the picture and word changed (both new). The number of trials is consistent with previous research using a similar paradigm in children (Sloutsky and Napolitano, 2003; Napolitano and Sloutsky, 2004; Robinson and Sloutsky, 2019) and in adults (Dunifon et al., 2016; Barnhart et al., 2018). See Figure 1 for an overview of the different trial types.

#### Delayed Recognition Task

The delayed recognition task was designed to examine the effects of sensory memory on auditory dominance. The task was identical to the immediate recognition task, with the exception that the ISI in the unimodal and bimodal tasks both increased from 1 to 4 s.

#### Working Memory Task

**FIGURE 2:**
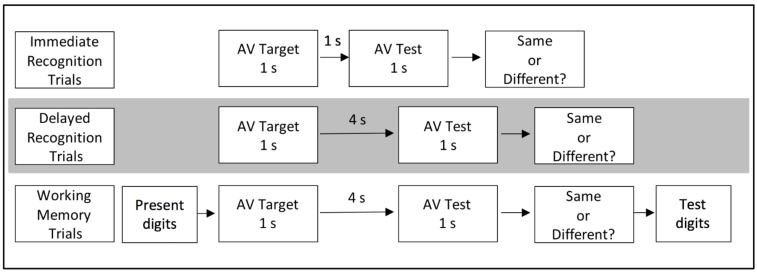
Overview of the immediate recognition, delayed recognition, and working memory tasks.

The working memory task was designed to examine the effects of verbal rehearsal on auditory dominance. To achieve this, a working memory task was added to the delayed recognition task. See Figure 2 for an overview of the three different bimodal tasks. For example, at the beginning of the unimodal working memory block, participants received the following instructions: *You will first be presented with a six-digit number. Rehearse and try to remember the number because you will be asked about the number at the end of the trial. After you see the number, you will either hear two words or see two pictures. You have to determine if the two words or two pictures are exactly the same or different*. On each trial, a different six-digit number was presented at the beginning of each trial for 1000 ms followed by a 1000 ms delay. Participants were then presented with a target for 1 s, followed by a 4 s ISI. At that point, a test item was presented for 1 s and the participant had to determine if the target and test were the same or different. Once the participant keyed in his/her response, a second six-digit number was presented on screen and the participant was instructed to respond whether the second six-digit number was the same or different from the original number. Participants were instructed to press 1 if the second six-digit number was the same and to press 3 if it was different. Half of the trials were same and half were different. Different numbers only varied by one number, and the changed number was equally likely to appear as the first digit, the fourth digit, etc. The experiment proper was identical to the Delayed Recognition task.

## Results

On each trial, participants had to determine if two words, two pictures, or two word-picture pairs were exactly the same or different. Participants did not miss any of the new auditory, new visual, or both new trials when presented unimodally or bimodally. Thus, we focused exclusively on response times across the different conditions. Moreover, we only analyzed response times on correct trials, and response times three standard deviations above the mean were also removed. Forty-seven, 50, and 61 trials were removed in the immediate recognition, delayed recognition, and working memory tasks, respectively, which was approximately 2.5% of the data. We begin by focusing exclusively on new auditory and new visual trials because sensory modalities on these trials are providing conflicting information, and therefore, these trials can provide information about modality dominance.

Response times were submitted to a 2 (Modality: Auditory vs. Visual) by 2 (Presentation: Unimodal vs. Bimodal) by 3 (Task: Immediate recognition vs. Delayed recognition vs. Working memory) repeated measures ANOVA. Note that Modality denotes which component changed at test. More specifically, “Auditory” is associated with response times on different trials where only the auditory component changed, and “Visual” is associated with response times on different trials where only the visual component changed. The analysis revealed a main effect of task, *F*(2,94) = 12.37, *p* < 0.001, $\eta_{p}^{2}$ = 0.21, with participants responding faster on immediate recognition trials (*M* = 929 ms, *SE* = 34) than working memory trials (*M* = 1120 ms, *SE* = 52), pairwise comparison with Bonferroni adjustment *p* < 0.001. Response times on delayed recognition trials (*M* = 962 ms, *SE* = 40) were also significantly faster than response times on working memory trials, *p* = 0.007. A main effect of modality was also revealed, *F*(1,47) = 33.53, *p* < 0.001, $\eta_{p}^{2}$ = 0.42, with response times being significantly faster on visual trials (*M* = 939 ms, *SE* = 39) than auditory trials (*M* = 1068 ms, *SE* = 35).

**FIGURE 3:**
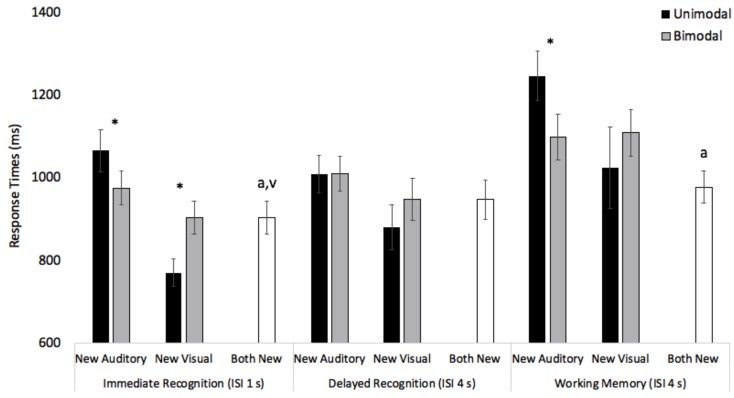
Response times across modality, presentation mode, and task for new auditory, new visual, and both new trials (*N* = 48). Error bars denote Standard Errors. Note that “^∗^” denotes that unimodal and bimodal response times differed, “a” denotes that both new response times were faster than unimodal auditory response times, and “v” denotes that both new response times were slower than unimodal visual response times, *p*s < 0.05.

The ANOVA also revealed a significant interaction between modality and presentation, *F*(1,47) = 34.11, *p* < 0.001, $\eta_{p}^{2}$ = 0.42, with bimodal presentation speeding up auditory responses and slowing down visual responses. In particular, conflicting visual information in the bimodal condition sped up auditory responses (*M* = 1029 ms, *SE* = 35) compared to the unimodal auditory baseline (*M* = 1107 ms, *SE* = 41), *t*(47) = 2.50, *p* = 0.016. In contrast, conflicting auditory information in the bimodal condition slowed down visual responses (*M* = 987 ms, *SE* = 38) compared to the unimodal visual baseline (*M* = 891 ms, *SE* = 49), *t*(47) = -2.52, *p* = 0.015. The analysis also revealed a marginally significant three-way interaction between task, modality, and presentation mode, *F*(2,94) = 2.48, *p* = 0.089, $\eta_{p}^{2}$ = 0.050. See black and gray bars in Figure 3 for means and standard errors across the different tasks. As can be seen in the figure, bimodal presentation significantly sped up auditory processing in the immediate recognition and working memory tasks, *t*s(47) > 2.06, *p*s < 0.045. In contrast, bimodal presentation slowed down visual responses in all three tasks, however, the slowdown only reached significance in the immediate recognition task, *t*(47) = -3.19, *p* = 003.

The analyses examining trials were only the auditory or visual stimuli changed at test are consistent with auditory dominance, however, we also examined response times on same trials and both new trials. While both of these trials provide redundant cross-modal cues in the bimodal condition (i.e., both stimuli are associated with a same response or a different response), task demands are different across these two trial types. For example, to be accurate on same trials, participants had to detect that both words are the same and also detect that both pictures are the same. Thus, even though there is intersensory redundancy, task demands increase on same trials, and response times could slow down compared to the unimodal baselines or be comparable to response times in the slower modality (assuming stimuli are processed in parallel). In contrast, on both new trials, participants can quickly respond “different” the moment they detect any change, with no need to process the other modality. Thus, under this scenario, it is possible that response times might speed up when presented bimodally (or be comparable to the faster modality). We begin by focusing on both new trials and then we focus on same trials.

To determine how bimodal redundant cues affected processing, we compared response times on both new trials with response times on unimodal trials where the word or picture changed. Response times were submitted to a 3 (Condition: Unimodal Auditory vs. Unimodal Visual vs. Bimodal) × 3 (Task: Immediate recognition vs. Delayed recognition vs. Working memory) repeated measures ANOVA. Unimodal auditory and unimodal visual response times are denoted by black bars in Figure 3, and both new response times are denoted by a white bar in Figure 3. The analysis revealed a main effect of task, *F*(2,94) = 38.18, *p* < 0.001, $\eta_{p}^{2}$ = 0.15. Pairwise comparisons with Bonferroni adjustment revealed that response times in the immediate recognition (*M* = 913 ms, *SE* = 36) and delayed recognition (*M* = 945 ms, *SE* = 41) tasks were significantly faster than in the working memory task (*M* = 1083 ms, *SE* = 57), *p*s < 0.035. The effect of condition was also significant, *F*(2,94) = 24.47, *p* < 0.001, $\eta_{p}^{2}$ = 0.34. Unimodal auditory response times (*M* = 1107 ms, *SE* = 41) were slower than unimodal visual responses (*M* = 891 ms, *SE* = 49) and bimodal responses (*M* = 942 ms, *SE* = 34), Bonferroni adjusted pairwise *p*s < 0.001.

The condition × task interaction was also significant, *F*(4,188) = 2.94, *p* = 0.022, $\eta_{p}^{2}$ = 0.06. As can be seen in Figure 3, response times on both new trials were significantly faster than unimodal auditory trials in the immediate recognition and working memory tasks, *t*s (47) > 4.16, *p*s < 0.001. In contrast, in the immediate recognition task, response times on both new trials were significantly slower than the unimodal visual trials, *t*(47) = -3.68, *p* = 0.001. Finding a slowdown on both new trials relative to unimodal visual trials suggests that interference effects are not simply associated with conflicting auditory cues slowing down visual responses. Redundant auditory cues also slowed down responses.

**FIGURE 4:**
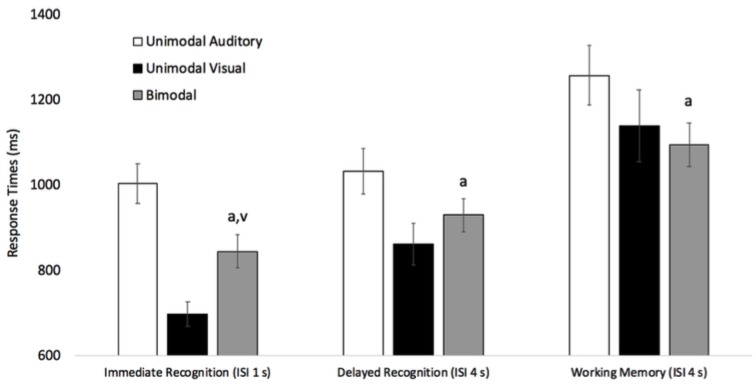
Response times across task and condition for same Trials (*N* = 48). Error bars denote Standard Errors. Note that “a” denotes that bimodal same response times were faster than unimodal auditory same response times, and “v” denotes that bimodal same response times were slower than unimodal visual same response times, *p*s < 0.05.

We also examined response times on same trials. Response times on same trials were submitted to a 3 (Condition: Unimodal Auditory vs. Unimodal Visual vs. Bimodal) × 3 (Task: Immediate recognition vs. Delayed recognition vs. Working memory) repeated measures ANOVA. Response times across condition and task are reported in Figure 4. The analysis revealed a main effect of task, *F*(2,94) = 30.14, *p* < 0.001, $\eta_{p}^{2}$ = 0.39. Pairwise comparisons with Bonferroni adjustment revealed that response times on immediate recognition (*M* = 849 ms, *SE* = 31), delayed recognition (*M* = 942 ms, *SE* = 40), and working memory (*M* = 1165 ms, *SE* = 55) tasks all significantly differed from each other, *p*s < 0.015. The effect of condition was also significant, *F*(2,94) = 17.63, *p* < 0.001, $\eta_{p}^{2}$ = 0.27. Unimodal auditory response times (*M* = 942 ms, *SE* = 40) were slower than unimodal visual (*M* = 942 ms, *SE* = 40) and bimodal (*M* = 942 ms, *SE* = 40) responses, Bonferroni adjusted pairwise *p*s < 0.001.

Finally, the condition × task interaction was also significant, *F*(4,188) = 2.53, *p* = 0.042, $\eta_{p}^{2}$ = 0.05. As can be seen in Figure 4, there was some evidence that same responses in the bimodal condition were slower than same responses in the unimodal visual condition, however, this effect was only significant in the immediate recognition task, *t*(47) = -4.42, *p* < 0.001. This pattern also replicates new auditory and new visual analyses (see Figure 3). In contrast, across all three tasks, same responses in the bimodal condition were faster than same responses in the unimodal auditory conditions, *t*s (47) = 2.16, *p*s < 0.036. Thus, participants were faster to indicate that two words and two pictures were the same, than they were to indicate that two words were the same.

## Discussion

Most of our experiences are multisensory in nature, and there is a growing body of research examining how sensory modalities interact while processing multisensory information (see Stein and Meredith, 1993; Bahrick et al., 2004; Calvert et al., 2004; Spence and Driver, 2004; Spence and McDonald, 2004; Talsma et al., 2010; Spence et al., 2012; van Atteveldt et al., 2014; Murray et al., 2016; Spence, 2018, for reviews). Simultaneously presenting information to multiple sensory modalities can sometimes facilitate learning and speed up responding (Miller, 1982; Giard and Peronnet, 1999; Bahrick and Lickliter, 2000; Bahrick et al., 2002; Fort et al., 2002; Colonius and Diederich, 2006; Sinnett et al., 2008), however, there are also many situations where stimuli in one modality interfere or alter perception in another modality (Colavita, 1974; McGurk and MacDonald, 1976; Shams et al., 2000, 2002; Sloutsky and Napolitano, 2003). The primary goal of this research was to examine possible mechanisms underlying cross-modal interference by examining how quickly participants discriminated auditory and visual information when presented in isolation and how task demands and bimodal presentation affect these speeded discriminations.

Participants in the current study had to quickly determine if two words, two pictures, or two word-picture pairings were the same or different, and we manipulated the time between the two stimuli (either 1 s or 4 s) and we also added a working memory task on some trials to block rehearsal of the auditory stimulus. When there was only 1 s separating the stimuli (immediate recognition task), auditory dominance was found with bimodal presentation slowing down visual processing and speeding up auditory processing. Increasing the interstimulus interval to 4 s and blocking verbal rehearsal weakened auditory dominance effects, however, conflicting visual information sped up auditory discriminations. Examination of new auditory and new visual trials (auditory input was associated with one response and visual input was associated with the opposite response), same trials (auditory and visual stimuli were both associated with a “same” response) and both new trials (auditory and visual stimuli were both associated with a “different” response) all provide corroborating evidence that bimodal presentation sped up auditory processing and slowed down visual processing.

Over the last 40 years, a considerable amount of research has pointed to visual dominance in adults, with participants quickly responding to the visual component of an auditory-visual pairing and often failing to respond to the auditory component (see Spence, 2009; Spence et al., 2012, for reviews). While the current study used a different task and relied on a different measure (response times associated with discriminating auditory and visual information, as opposed to types of errors made on bimodal trials), the current study provides some support for auditory dominance in adults. More specifically, simultaneously presenting auditory and visual information slowed down visual responses and often sped up auditory responding. While it is possible that effects on traditional Colavita tasks and the current task are qualitatively different in nature, the current findings suggest that auditory information disrupts visual processing, whereas, there was no evidence suggesting that visual information disrupts auditory processing.

To account for this asymmetry, Robinson and Sloutsky (2010a) suggested that auditory and visual stimuli may be competing for attention, with auditory stimuli often winning the competition due to the dynamic and transient nature of these stimuli. While these low-level effects could account for the finding that bimodal presentation is more likely to disrupt visual processing, these effects could also stem from the stronger memory trace dominating the less robust memory trace. For example, auditory stimuli persist longer in sensory memory than visual stimuli (Sperling, 1960; Darwin et al., 1972; Lu et al., 1992), thus, when participants are determining if a presented item matches one stored in memory, there may be an auditory advantage if the auditory stimulus has a stronger memory trace. It is also possible that participants can maintain the auditory stimulus in memory by using verbal rehearsal (Baddeley and Hitch, 1974), whereas, it may be more difficult to keep unfamiliar images in working memory. The primary goal of the current research was to potentially eliminate high-level explanations by increasing the interstimulus interval (delayed recognition task) and by blocking rehearsal of the words (working memory task).

On the surface, increasing the ISI to 4 s appeared to attenuate auditory dominance effects, as there was no significant slowdown in visual processing in the bimodal condition compared to the unimodal visual baseline. However, this attenuation appeared to be driven by a change in discriminating the unimodal visual stimuli, not in eliminating interference (i.e., speeding up bimodal responses). For example, as can be seen in Figure 3, increasing the ISI from 1 to 4 s slowed down unimodal visual response times, whereas, the increase in ISI had less of an effect on auditory processing. This may suggest that increasing the ISI to 4 s was enough time for the visual but not auditory trace to fully decay from sensory memory. Moreover, consistent with the immediate recognition task in the current experiment, there appears to be a visual response time advantage when discriminating unimodal stimuli with a relatively short ISI (Dunifon et al., 2016; Barnhart et al., 2018) and it is possible that auditory interference effects may be more pronounced when there is initially a visual response time advantage. Some support for this claim comes from children, young adults, and older adults in Barnhart et al. (2018). More specifically, children and young adults in Barnhart et al. (2018) showed evidence of auditory dominance, and they were also faster at discriminating the unimodal visual stimuli compared to the unimodal auditory stimuli. Older adults were faster at discriminating the unimodal auditory stimuli, and the pattern reversed, with bimodal presentation only interfering with auditory processing. Thus, it is possible that some of the modality dominance effects stem from bimodal presentation having a greater cost on processing in the faster modality and sometimes speeding up processing in the slower modality. If this account is correct, it should be possible to predict the direction of modality dominance effects by knowing unimodal response times, with multisensory presentation having a greater cost on processing in the faster modality.

Perhaps the most interesting result of the current study was the finding that adding visual information to the task often sped up auditory responding. For example, across all three tasks, analysis of same trials showed faster responding in the bimodal condition compared to the unimodal auditory condition. Facilitation effects in the bimodal condition were also significant in two of the three tasks examining new auditory trials where visual information provided conflicting information. While speculative, it is possible that bimodal presentation made the auditory task more engaging and the faster response times simply reflect increased arousal. This general increase in arousal would explain why effects were found on both conflicting and redundant trials and may also explain why the facilitation effects on new auditory trials only reached significance on immediate recognition and working memory tasks (see Figure 3). The immediate recognition task is a relatively fast procedure with a lot of information to encode and store in a short time and the working memory task increased load and required participants to stay engaged throughout the entire trial. In contrast, the delayed recognition task was slow paced, with a 4 s ISI on each trial, and this task was less likely to result in facilitation/interference effects.

While the current study sheds light on modality dominance effects, there are some potential limitations. First, it is difficult to equate for familiarity, complexity, and saliency across sensory modalities. For example, it could be argued that attenuated visual processing in the current study resulted from task difficulty, not because of auditory interference. While this is possible given that increased demands across tasks appeared to have a greater cost on unimodal visual discrimination, this seems unlikely because unimodal visual response times were faster across all three tasks suggesting that visual discrimination may have actually been easier.

We also tried to partially address these modality difference issues by avoiding direct comparisons between auditory and visual information. Recall that the current study examined processing speed when stimuli were presented unimodally with processing speed when the same stimuli were presented with a stimulus from a different sensory modality. Second, sensory memory studies often use stimulus durations that are relatively short in duration. For example, in Sperling (1960), visual stimuli were only presented for 50 ms, whereas, they were presented for 1 s in the current study. We could not significantly decrease stimulus duration in the current study because 50 ms is not long enough to present spoken words, thus, future research examining differential decay in sensory memory will also need to change the class of auditory stimuli and examine modality dominance effects under shorter durations. Finally, the means in the current study were computed by averaging across six trials. This is consistent with previous research (Sloutsky and Napolitano, 2003; Napolitano and Sloutsky, 2004; Dunifon et al., 2016; Barnhart et al., 2018), however, future research will need to increase the number of trials to decrease error variance.

In summary, the cognitive processes underlying intersensory interactions are complex and not fully understood. The current study contributes to this research by examining how pairing words and sounds together affect the speed of auditory and visual processing. Consistent with previous research (Dunifon et al., 2016; Barnhart et al., 2018), pairing pictures and words together appeared to slow down visual response times, while having no cost on auditory processing. While the same general trend was found across all conditions, these effects were most pronounced when there was a relatively short time between compared stimuli and possibly because there was initially a visual response time advantage. These findings are consistent with a potential mechanism underlying auditory dominance, which posits that sensory modalities are competing for attention, with auditory stimuli often winning the competition due to the dynamic and transient nature of these stimuli (Robinson and Sloutsky, 2010a). Finally, pairing the pictures and words together appeared to have a different effect on auditory processing, with the additional visual information, even conflicting visual information, speeding up auditory processing. These findings have implications on many tasks that hinge on simultaneous processing of auditory and visual information.

## Ethics Statement

This study was carried out in accordance with the recommendations of Behavioral and Social Science Institutional Review Board at The Ohio State University, with written informed consent from all subjects. All subjects gave written informed consent in accordance with the Declaration of Helsinki. The protocol (2014B0022) was approved by the Behavioral and Social Science Institutional Review Board at The Ohio State University.

## Author Contributions

CR, RM, and TC designed the study, created stimuli, and collected/analyzed the data. TC wrote up the first draft of the manuscript.

## Conflict of Interest Statement

The authors declare that the research was conducted in the absence of any commercial or financial relationships that could be construed as a potential conflict of interest.
